# DNA Barcode Authentication of Wood Samples of Threatened and Commercial Timber Trees within the Tropical Dry Evergreen Forest of India

**DOI:** 10.1371/journal.pone.0107669

**Published:** 2014-09-26

**Authors:** Stalin Nithaniyal, Steven G. Newmaster, Subramanyam Ragupathy, Devanathan Krishnamoorthy, Sophie Lorraine Vassou, Madasamy Parani

**Affiliations:** 1 Department of Genetic Engineering, Center for DNA Barcoding, SRM University, Chennai, India; 2 Interdisciplinary School of Indian System of Medicine, SRM University, Chennai, India; 3 Centre for Biodiversity Genomics, University of Guelph, Guelph, Ontario, Canada; Max F. Perutz Laboratories, Austria

## Abstract

**Background:**

India is rich with biodiversity, which includes a large number of endemic, rare and threatened plant species. Previous studies have used DNA barcoding to inventory species for applications in biodiversity monitoring, conservation impact assessment, monitoring of illegal trading, authentication of traded medicinal plants etc. This is the first tropical dry evergreen forest (TDEF) barcode study in the World and the first attempt to assemble a reference barcode library for the trees of India as part of a larger project initiated by this research group.

**Methodology/Principal Findings:**

We sampled 429 trees representing 143 tropical dry evergreen forest (TDEF) species, which included 16 threatened species. DNA barcoding was completed using *rbcL* and *matK* markers. The tiered approach (1^st^ tier *rbcL*; 2^nd^ tier *matK*) correctly identified 136 out of 143 species (95%). This high level of species resolution was largely due to the fact that the tree species were taxonomically diverse in the TDEF. Ability to resolve taxonomically diverse tree species of TDEF was comparable among the best match method, the phylogenetic method, and the characteristic attribute organization system method.

**Conclusions:**

We demonstrated the utility of the TDEF reference barcode library to authenticate wood samples from timber operations in the TDEF. This pilot research study will enable more comprehensive surveys of the illegal timber trade of threatened species in the TDEF. This TDEF reference barcode library also contains trees that have medicinal properties, which could be used to monitor unsustainable and indiscriminate collection of plants from the wild for their medicinal value.

## Introduction

India is the custodian for considerable biodiversity as it intersects four global biodiversity hotspots, and is the eighth largest country among the 17 mega biodiversity countries [1]. According to the India’s fourth report to the convention on biological diversity, it harbors nearly 11% of the world’s floral diversity, which includes ca. 6000 endemic species and over 246 globally threatened species [2]. India’s biogeography is diverse with ten different bio-geographic zones, of which 23.4% of the land area is forested [3], [4]. The forests in India are classified into 6 major types and 16 minor types on the basis of structure, physiognomy and floristic diversity [5]. The tropical dry evergreen forest (TDEF) is one of the minor forest types classified within the major forest type, tropical dry forest (TDF). The TDEF of India is part of the costal bio-geographic zone that is narrowly confined to the East coast, which is under considerable development pressures. Tropical forest ecosystems are known as critical habitats for the conservation of biodiversity, and these ecosystems are threatened by urbanization and climatic change resulting in species extinction at the rate of 0.8% to 2% per year [6]. The TDEF in India is particularly vulnerable because of its very narrow geographic boundaries. The forest cover under TDEF is rapidly declining due to overexploitation for timber, fuel wood, and construction of infrastructure such as buildings, dams, and roads. This has recently resulted in substantial media calling for conservation measures within the TDEF of India. Notable scientists have reported that the impending threat to the rich native biodiversity in the TDEF of India is partly due to its inherent abundance in natural resources [7], [8]. The TDEF needs to be given high priority for natural resource planning strategies that conserve biodiversity as envisioned in National Environment Policy [9].

Quick and reliable species identification is needed in order to facilitate the large-scale biodiversity inventories required for conservation strategies [10]. Taxonomic identification of tropical trees can be challenging; individual trees of a species may vary morphologically according to their age and growing conditions, and at the same time, closely related species can look morphologically similar [11]. Traditional taxonomic methods based on morphological identifications are costly and require a considerable amount of time in order to provide accurately identified plants [12]–[15]. There are only a few taxonomists in India with botanical field experience who can reliably identify all the tree species in TDEF. Moreover, it is extremely difficult to identify the species when the specimen is incomplete, damaged or derived from plant parts such as leaves, roots, bark, wood and seeds. It is desirable to utilize an alternate method for species identification that can use specimens in different forms (e.g., wood) and life stages. Recent advances in DNA sequencing and molecular diagnostic tools for plants [16], [17] have the capacity to improve upon traditional methods of species identification [18].

DNA barcoding is emerging as a valuable tool for quick assessments of biodiversity that provides high quality data for developing conservation strategies [19], [20]. A recent study reported assessment data from the same site wherein DNA barcoding survey provided more accurate estimates (42% more species) than traditional morphological taxonomic survey, which was 37% more expensive than barcoding [21]. DNA barcoding uses a short standardized DNA sequence for species identification that is divergent between species but conserved within species [22]. While cytochrome c oxidase I (COI) gene is widely regarded as a universal DNA barcode to identify most groups of animals, a different approach has been taken for plants. This is due to the fact that there is little COI variation in plants and there has been difficulty in identifying a single universal barcode marker for plants; plants have inherently low nucleotide variation in recently evolved species, and undergo complex evolutionary processes such as hybridization and polyploidy [23], [24]. Although many researchers have searched for a single region for barcoding plants, it is generally agreed that a multi-locus barcode combination would be required to discriminate plant species [25]–[29]. Newmaster *et al.* [17] and Purushothaman *et al*. [30] described this as the multigene tiered approach wherein barcodes are constructed from two ‘tiered’ gene regions; an easily amplified and aligned region is used for the first tier (*rbcL*) that acts as a scaffold on which data from a more variable second-tier region are interpreted for species identification. The chloroplast *rbcL* was proposed as the first tier marker because of its universality and demonstrated success for differentiating congeneric plant species [17], [31]. The second tier variable marker may be chloroplast *trnH*-*psbA* (non-coding) and *matK* (coding) or nuclear ITS2.

DNA barcoding has been used in many botanical studies ranging from detailed study on single genus to ecosystem level surveys in tropical, subtropical and temperate forests. DNA barcoding of all the 1073 trees in two hectares of a tropical forest in French Guiana showed that it could increase the quality and the speed of biodiversity surveys [13]. It was found to be useful for detecting errors in morphological identifications and increased the identification rate of juveniles from 72% to 96%. DNA barcoding of 200 accessions from two 0.1 hectare tropical forest plots in Northeast Queensland also showed that it could rapidly estimate species richness in forest communities [12]. Tripathi *et al*. [32] have studied 300 specimens from tropical trees of North India, and suggested that DNA barcoding will be useful in large-scale biodiversity inventories. Vegetation surveys in four equally sized temperate forest plots in the Italian pre-alpine region of Lombardy, Valcuvia by morphological identification and DNA barcoding revealed that the later could save time and resources [18]. Parmentier *et al*. [11] have assessed the accuracy of DNA barcoding in assigning a specimen to a species or genus by studying 920 trees from five lowland evergreen forest plots in Korup and Gabon, Africa. DNA Barcoding was found to be useful in assigning unidentified trees to a genus, but assignment to a species was less reliable, especially in species-rich clades. In a large study that included 2,644 individuals representing 490 vascular plant species, mostly from the Canadian Arctic zone, again showed that DNA barcoding differentiated the taxa more at the genus level than at the species level [33].

In another interesting study of tropical forest, DNA barcoding was applied on 1,035 samples representing all the 296 species of a Forest Dynamics Plot on Barro Colorado Island in Panama [34]. Barcode data from *rbcL*, *matK* and *trnH*-*psbA* were found to be sufficient to reconstruct evolutionary relationships among the plant taxa that were congruent with the broadly accepted phylogeny of flowering plants. The same research group studied another Forest Dynamics Plot in the Luquillo Mountains of Northeast Puerto Rico that encompassed a mix of old growth and secondary forest that has been largely free from human disturbance since the 1940 s. This study again reinforced the congruence of the barcode phylogeny with the phylogeny of flowering plants as per APG III classification [35]. DNA barcoding was also used to construct community phylogeny in order to understand the patterns of species occurrence in forest habitats [36]. Community phylogeny which was constructed for the Dinghushan Forest Dynamics Plot in China by sequencing *rbcL*, *matK,* and *trnH*-*psbA* loci from 183 species showed that closely related species tend to prefer similar habitats. The patterns of co-occurrence within habitats are typically non-random with respect to phylogeny. While phylogenetic clustering was observed in valley and low-slope, phylogenetic over-dispersion was characteristic of high-slope, ridge-top and high-gully habitats.

Our study reports DNA barcoding of tree species from the TDEF in India. The specific objectives of this project are to 1) Develop a TDEF reference barcode library for 143 tropical tree species, 2) Utilize the TDEF reference barcode library for species identification of lumber from logged timber sites, 3) To monitor the endemic and threatened species in timber trade, and 4) To prevent indiscriminate collection of non-timber forest products. This research seeks to provide a DNA reference barcode library for floristic assessments of tropical dry evergreen forests in biodiversity rich countries like India, which can be utilized for the conservation of rare and native tree species.

## Materials and Methods

### Sample collection

**Figure 1:**
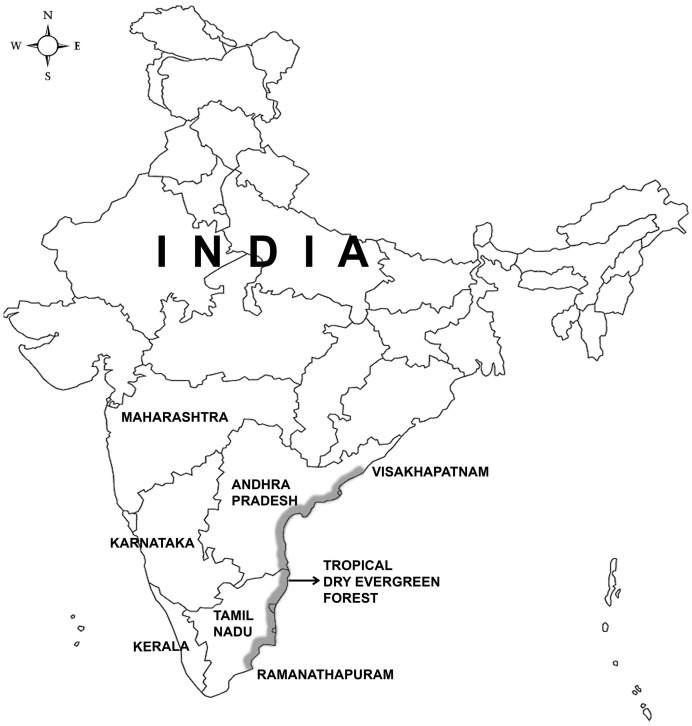
Map of India showing the distribution of Tropical Dry Evergreen Forest (*Painted in green colour) distributed between Visakhapatnam in Andhra Pradesh State and Ramanathapuram in Tamil Nadu State.

Our study area was the Tropical Dry Evergreen Forest (TDEF) of India, which is part of the costal bio-geographic zone. It is narrowly confined to the East coast (9° 22′ –17° 36′ N latitude and 78° 49′ –82° 56′ E longitude) between Visakhapatnam in Andhra Pradesh and Ramanathapuram in Tamil Nadu (Figure 1). The forests have three sub-classifications: sandy coast, interior coastal plains with red lateritic soil, and isolated hillocks wherein dense forest thickets are formed with evergreen and deciduous small trees and thorny shrubs. The TDEF receives an annual rainfall of 900 mm to 1200 mm. Depending on the geographical location, the dry season may extend from January to March or from December to May [37].

We sampled 429 trees representing 143 species (114 genera, 42 families and 19 orders) from different sites within TDEF, and their GPS coordinates are provided in Table S1. Out of the 143 tree species collected, 16 species are on the IUCN red list of threatened species as searched in the website http://www.redlist.org. All the samples were collected for research purpose only from cultivated sources, gardens and open forests which are accessible to any public, hence no permission was required. Voucher specimens from all the collections were professionally identified using local floras. They were mounted on standard herbarium sheets, and were deposited to the SRM University Herbarium. Leaves from each accession were air-dried, stored at room temperature, and later used for DNA extraction and barcoding. In addition, sap woods from 25 freshly logged trees were collected from timber shops at five different locations.

### DNA isolation

Genomic DNA was isolated by following the protocol of Saghai-Maroof *et al.* [38] with minor modifications. About 100 mg of leaf tissue was taken for genomic DNA isolation and ground using mortar and pestle by adding 500 µl of CTAB buffer (100 mM Tris-HCl, 1.4 M NaCl, 20 mM EDTA, 1% beta-mercaptoethanol, 2% CTAB). The samples were transferred to 1.5 ml centrifuge tubes, incubated in water bath at 55°C for 30 minutes, and then extracted with equal volume of chloroform. The samples were centrifuged at 10,000 rpm for 10 minutes, and the aqueous phase was transferred to fresh 1.5 ml centrifuge tubes. The DNA was precipitated by adding equal volume of ice-cold isopropanol, and centrifuged at 10,000 rpm for 10 minutes. The DNA pellet was washed with 70% ethanol, air-dried at room temperature, and dissolved in 100 µl TE buffer. In case of wood samples, genomic DNA was isolated by following the same protocol except that 2% PVP was included in the CTAB buffer, and the samples were incubated at 55°C for 10 hours.

### PCR amplification and DNA sequencing

PCR amplification of DNA barcode markers was done using 50 ng of total genomic DNA as template and the commonly used primers for *matK* (*matK*-1RKIM-F and *matK*-3FKIM-R, Ki-Joong Kim, School of Life Sciences and Biotechnology, Korea University, Korea, unpublished), and *rbcL* (*rbcLa*-F, *rbcLajf*634-R) [39], [40]. PCR reaction mixture (30 µl) contained 1X buffer with 1.5 mM MgCl_2_, 200 µM dNTPs, 5 pmol primers, and 1 unit *Taq* DNA polymerase. PCR was done in a thermal cycler (Eppendorf, Germany) using the following protocol: initial denaturation at 95°C for 5 minutes, 30 cycles of denaturation at 95°C for 30 seconds, annealing at 55°C for 30 seconds, and extension at 72°C for 1 minutes, final extension at 72°C for 5 minutes, and hold at 16°C. The PCR products were checked by agarose gel electrophoresis, and purified using EZ-10 Spin Column PCR Purification Kit (Bio Basic Inc. Ontario, Canada). The purified PCR products were sequenced from both ends using the same PCR primers in 3130×l Genetic analyzer (Applied Biosystems, CA, USA). The sequences were manually edited using Sequence Scanner Software v. 1.0 (Applied Biosystems, CA, USA) and full length sequences were assembled.

### Data analyses

The fully edited sequences with original trace files for *rbcL* and *matK* markers were submitted to Barcode of Life Database (BOLD Systems v.3.) under the project name “TDEF Project 1″ with process IDs TDEF001-12 to TDEF429-12. The details of the 429 samples that were used in the present study, their process IDs in BOLD database, PCR success and length of *rbcL* and *matK* sequences obtained are given in Table S2. These sequences were also used to create a TDEF reference barcode library. Pairwise divergence was calculated in BOLD Systems v. 3 using Kimura 2 parameter distance model and MUSCLE program [41]. Database search for species identification were done using Basic Local Alignment Search Tool (BLAST) against non-redundant nucleotide database at NCBI (www.blast.ncbi.nlm.nih.gov/Blast.cgi). We assessed the species resolution of the two DNA barcodes using three different methods; the best match method [42], phylogenetic method [43], and Characteristic Attribute Organization System (CAOS) [44].

Best match method for species identification was carried out using TaxonDNA version 1.6.2, [42] which is available at http://taxondna.sf.net/. In this method, each sequence was queried against TDEF reference barcode library to identify the species associated with its closest match based on the genetic distance. The query identification was considered a “success” when the two sequences were from the same species, “ambiguous” when it matched with more than one species at the same genetic distance, and “failure” when the two sequences were from mismatched species.

Phylogenetic tree was constructed after combining the *rbcL* and *matK* barcode sequences. Genetic distances were calculated by K2P distance model and phylogenetic trees were constructed by Neighbor-Joining (NJ) method using ClustalW in MEGA v. 5.1 [43]. Bootstrap support was analyzed with 1,000 replications. All positions containing gaps and missing data were eliminated from the analysis. Species were distinguished based on genetic distance and monophyly.

Characteristic Attribute Organization System (CAOS) was used to identify diagnostic characteristic attributes (CAs) for species identification [44], [45]. Sequence data matrix and tree file were generated using the program MESQUITE v. 2.6 [46]. The resulting NEXUS file which consists of a non-interleaved DNA data matrix, a translate block (converts the taxon names to higher values in the tree representation) and a Newick tree file with collapsing nodes relative to the taxonomic groupings of interest was used in CAOS in accordance with the manual (www.boli.uvm.edu/casos-workbench/manual). First, it was used in the P-Gnome program to determine diagnostic positions at each major taxonomic grouping. Then, new sequences were classified into taxonomic groupings using the P-Elf program. Finally, the most variable sites that distinguish all the taxa were chosen. The character states at these nucleotide positions were listed and unique combinations of CAs were identified.

## Results and Discussion

### PCR amplification and bidirectional sequencing of *rbcL* and *matK* markers

Success of PCR amplification and sequence recoverability is an important criterion for assessing the utility of DNA barcodes. In our study, *rbcL* and *matK* barcode markers were amplified using universal primer pairs and standard protocols for most of our samples, despite the fact that these plant samples represented 42 diverse families. The *rbcL* marker was successfully amplified from all the samples, whereas the *matK* marker was amplified only in 75.8% of the samples. There was no variation in sequence length for *rbcL*; bidirectional sequencing recovered the 607 bp target sequence for all the PCR amplicons. Bidirectional sequencing was successful in 98% of the *matK* PCR amplicons, and there was considerable variation in the sequence length. Length of the *matK* sequence (Q value >40) varied between 508 bp and 867 bp with an average of 803 bp (500 bp is acceptable for the submission to BOLD database). Our results support earlier studies that report no variation in sequence length for *rbcL* along with high PCR amplification and sequencing success [25], [47], which in some studies reaches 100% [48], [49]. Previous researches suggest that *matK* PCR success rate is highly variable, ranging from 40% to 97% [39], [48]. Although we did not record any repeat sequences in *matK* as documented in other studies [50] in which it impacted the sequencing quality and success; repeat sequences in *matK* are not as common as those found in *trnH-psbA* [51].

### Intra/inter-specific divergence

Intra-specific and inter-specific divergence are useful for assessing DNA barcodes [29], [52], [53]. We calculated divergence among the individuals of the same species (intra-specific divergence) as well as the species of individual genus (inter-specific divergence) wherever multiple species in a genus where included in the study. Intra-specific divergence varied from 0.0% to 0.33% and 0.0% to 0.49% for *rbcL* and *matK*, respectively. Inter-specific divergence varied from 0.0% to 1.8% for *rbcL*, and 0.0% to 2.6% for *matK.* Our study included 44 congeneric species from 15 genera for which pairwise divergences were considered for their ability to differentiate the species. The number of congeneric species per genus varied between 2 and 7 species, and they formed 63 congeneric species pairs. Data from *rbcL* was available for all the pairs, and it differentiated 28 (44%) species when cut-off for intra-specific divergence was set at 0.5% (Table S3). At this cut-off level, *matK* differentiated 35 (92%) species (Table S4). We defined barcoding gaps as the difference between minimum inter-specific and maximum intra-specific divergence, as calculated for the congeneric species. Barcoding gap was observed in 11 genera, and it varied from 0.16% to 0.66% and 0.38% to 1.55% for *rbcL* and *matK* marker, respectively. In general, the barcoding gap is narrow due to the existence of closely related congeneric species. There was a large overlap between intra-specific and inter-specific pairwise distances among the congeneric species of deciduous trees of which the observed barcoding gap ranged between 0.2% and 0.9% [54]. Comparable levels of the barcoding gap were reported in *Agalinis* that ranged between 0.44% and 0.76% [55]. If pairwise divergence across all the species (non-congeneric) is considered, *rbcL* and *matK* differentiated 45.14% and 90% of the species, respectively. Previous researches have reported *matK* to have only slightly more discriminatory power than *rbcL* [27], [28]. We report a considerably larger difference, but this may be attributed to the fact that 24% of our samples are from Fabaceae, and *matK* was shown to have more than 80% species differentiation in this family [56].

### Barcode species resolution

It is estimated that the TDEF in India has ca. 1,500 species of which ca. 300 species are trees. Therefore, the TDEF represents about 11.5% of the 2,560 tree species found in India [57]. We have generated TDEF reference barcode library for the first time with 429 *rbcL* and 318 *matK* barcodes that were derived from 143 tree species.

### Best match method for species ID

The best match method is the simplest method for species identification [42]. It assigns the query sequence to a species with which it shows the smallest genetic distance. The *rbcL* and *matK* barcode sequences from individual samples were queried against sequences in the TDEF reference barcode library. The *rbcL* marker correctly identified 129 out of 143 species (90.2%) with the smallest genetic distance among all the species. Species identification for the remaining samples was ‘ambiguous’ because they showed same genetic distance with more than one species. The *matK* marker correctly identified the samples from 113 out of 117 species (96.5%). The strict combined marker (*rbcL*+*matK*) approach correctly identified the samples from 115 out of 117 species (98.3%) (Table 1). The tiered approach (1^st^ tier *rbcL*; 2^nd^ tier *matK*) correctly identified the samples from 136 out of 143 species (95%). The distance based methods have been criticized because it is extremely difficult to determine a single universal threshold genetic distance for distinguishing taxonomic groups [58], [59]; this is supported by the fact that the barcode gap can vary greatly across the groups [60]. Assigning group-specific thresholds either by following the “10X rule” of Herbert *et al.* [61] or otherwise is also not reliable when the estimated intra-group divergence does not represent the entire range of the distribution.

**Table 1:** Performance of DNA barcodes in sequence recovery and species identification success.

	Sequence recovery		Species identification success		
Barcodes	No. of accessions	No. of species	Best match method	Phylogenetic method	CAOS method
*rbcL*	429	143	129 (90.2%)	129 (90.2%)	129 (90.2%)
*matK*	318	117	113 (96.5%)	112 (95.7%)	113 (96.5%)
*rbcL*+*matK*	351	117	115 (98.3%)	115 (98.3%)	115 (98.3%)

### Phylogenetic method for species ID

**Figure 2:**
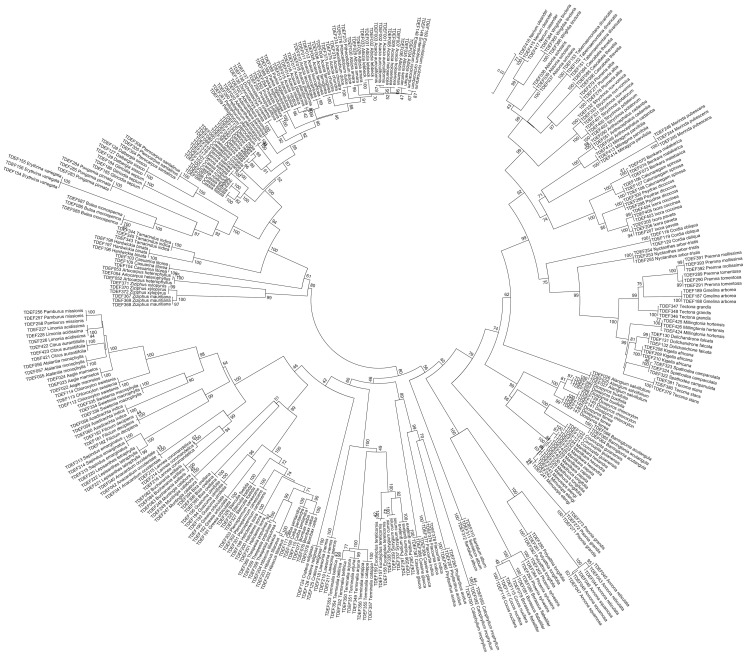
NJ tree of TDEF reference barcode library for *rbcL*+*matK* marker from 117 tree species.

**Figure 3:**
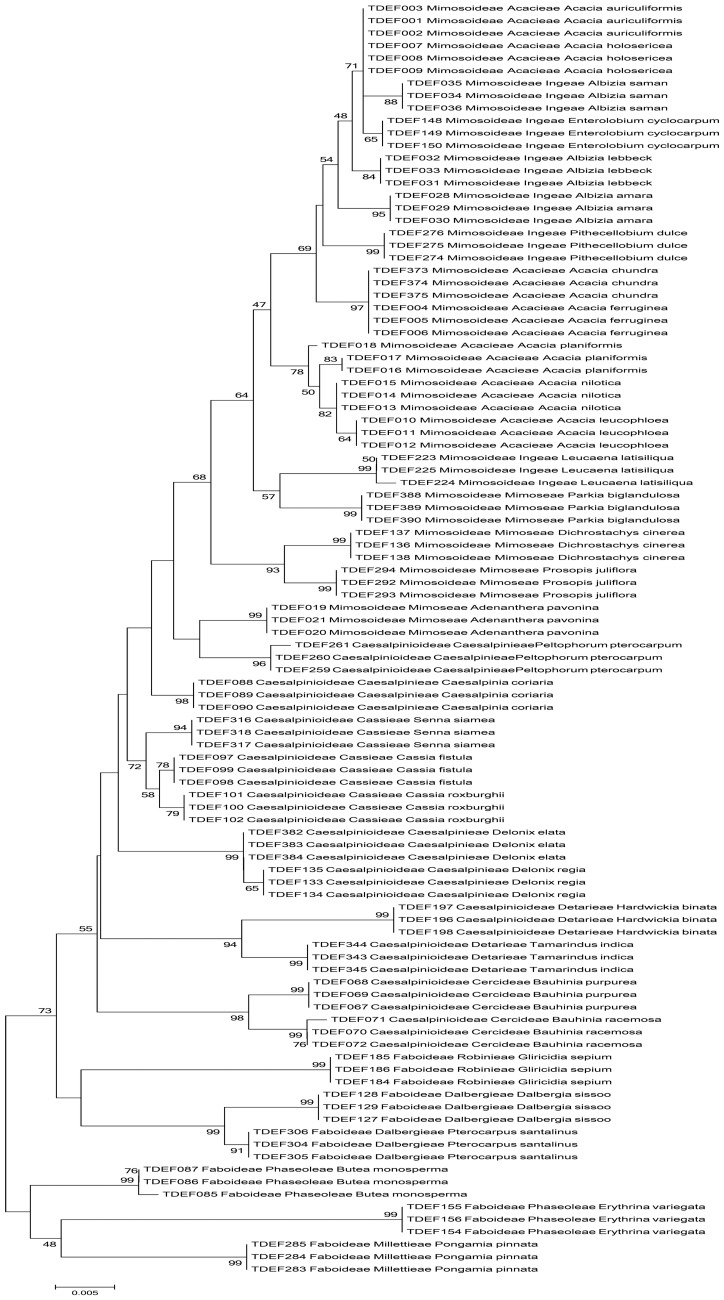
NJ tree of Fabaceae in the TDEF reference barcode library for *rbcL*+*matK* marker.

Phylogenetic tree based analyses are useful for evaluating discriminatory power by calculating the proportion of monophyletic species. A monophyletic clade includes the ancestor and all of its descendants that can be identified by the ability to remove it from the rest of the phylogenetic tree with a single cut. In our study, we constructed phylogenetic tree using the neighbor-joining method, which has been adopted by many floristic barcoding studies [33], [62]. Combined data for both *rbcL* and *matK* marker was available for 117 species belonging to 34 families. In the phylogentic tree, 30 families formed monophyletic groups, and 27 of them had bootstrap value between 70% and 100%. (Figure 2). The largest family that we studied was the Fabaceae, which included 23 genera and 34 species. Among the three subfamilies in Fabaceae, *Faboideae* was monophyletic while *Caesalpinioideae* was paraphyletic with respect to *Mimosoideae* (Figure 3). This is supported by the earlier phylogenetic report based on *rbcL* sequences as well as morphological characters [63]–[65]. Among the four tribes studied in *Faboideae, Dalbergieae* and *Robinieae* were monophyletic while *Millettieae* and *Phaseoleae* were not monophyletic (Figure 3). Polyphyly relationship between *Millettieae* and *Phaseoleae* was reported before based on morphological characters [66], chloroplast *rbcL* sequences [67], and nuclear phytochrome gene sequences [68]. *Caesalpinieae* in *Caesalpinioideae* as well as *Acacieae* and *Mimoseae* in *Mimosoideae* were not monophyletic (Figure 3). Earlier studies based on morphological as well as *rbcL* data have shown that *Mimoseae* is paraphyletic [69], [70]. In the genus level, all except *Acacia* and *Albizia* formed monophyletic groups. The non-monophyletic clade formed two branches: one branch contained only the species of *Acacia*; the other branch was shared by the species of *Acacia, Albizia*, *Enterolobium* and *Pithecelobium.* While *Acacia* belongs to tribe *Acacieae*, *Albizia*, *Enterolobium* and *Pithecelobium* belong to tribe *Ingeae.* Based on *matK* and *trnK* chloroplast sequences, it has been reported that the genus *Acacia* is not monophyletic [71], [72]. We also found a non-monophyletic clade outside the Fabaceae that was formed by *Pamburus* and *Aegle,* which belong to tribe *Aurantieae* of Rutaceae.

Our phylogenetic trees can also be used for differentiating species. We could differentiate 90.2%, 95.7%, and 98.3% of the species from the tree constructed using *rbcL*, *matK*, and *rbcL*+*matK*, respectively (Figure 2, Figure S1, Figure S2, and Table 1). The species that could not be differentiated based on *rbcL* marker included four species of *Acacia*, six species of *Ficus* and two species of *Annona*. In addition, the monotypic species *Aegle marmelos* could not be differentiated from *Pamburus missionis.* However, *matK* differentiated the two species each from *Acacia* and *Annona*, *P. missionis* and *A. marmelos* that could not be differentiated by *rbcL*. *Manilkara hexandra*, *M. zapota and Madhuca longifolia* of Sapotaceae were not distinguished by *matK* but were distinguished by *rbcL* albeit with very low genetic distance. It is reported that plastid markers perform poorly in recovering monophyletic species in Sapotaceae [13]. However, by combining the data from *rbcL* and *matK*, we could differentiate all except two species (*Acacia chundra* and *A. ferruginea*). Phylogenetic tree based methods have been criticized because they are not able to make use of low level of divergence, which is sufficient for differentiating groups but not for building phylogenetic relationships [33], [60].

### Characteristic attribute organization system (CAOS) method for species ID

The CAOS method identifies a combination of characteristic attributes (CAs) that is diagnostic to a particular group [73]. This method is based on the concept that members of a taxonomic group share characteristic attributes (CAs) that are absent in comparable groups. The CAOS algorithm thus identifies CAs for every clade at each branching node within a guide tree that is first produced from a data set. The resulting combination of diagnostics CAs can be used for subsequent classification of new data into the taxonomic groupings represented by the guide tree [73], [74]. This method has been used for DNA barcoding in animals [75], [76] and plants [55], [77]. Here we have employed CAOS method using *rbcL* and *matK* markers as character states, and stringently considered only single pure CAs (sPu), which are present in all member of one clade but absent in the other clades. We have found at least one sPu in 90.2%, 96.5%, and 98.3% of the species with *rbcL*, *matK* and *rbcL*+*matK*, respectively (Table 1). The number of sPu in individual species varied from 1 to 25 (average 6.5) and 1 to 58 (average 18) for *rbcL* and *matK*, respectively (Table 2, Table S5 and Table S6).

**Table 2:** Number of diagnostic characters (sPu) for TDEF tree species from *rbcL* and *matK* markers.

S.No	No. of sPu	No. of species	
		*rbcL*	*matK*
1	0	14	4
2	1–5	67	20
3	6–10	37	22
4	11–15	17	16
5	16–20	6	17
6	21–25	2	8
7	26–30	0	5
8	31–35	0	5
9	36–40	0	6
10	41–45	0	7
11	46–50	0	3
12	51–55	0	2
13	55–58	0	2

### Accuracy and applications of the TDEF reference barcode library

Species resolution from our study within the TDEF in India was 90.2% (*rbcL*) and 96% (*matK*) as estimated using three different methods of analysis. This estimate is much higher when compared with less than 72% species discrimination that is generally reported for *rbcL* and *matK* markers at a global scale [27], [28], but very similar to studies at a regional scale [20]. The high species resolution estimates from our study is likely attributed to the fact that the current TDEF reference barcode library is made of highly diverse species; 143 species representing 114 genera, and 42 (36.8%) of them are monotypic to the TDEF in India. In general, approximately 20% of the species in the TDEF in India are monotypic. Similar results were reported when DNA barcoding were applied on a regional scale in Barro Colorado Island of Panama and Northeast Puerto Rican forest [34], [35] and tropical rain forest of French Guiana [13]. Although the standard barcode markers recommended by CBOL were sufficient to resolve most of the species, we suggest the addition of a supplementary marker such as ITS2 to increase species resolution based on evidence from other studies [21], [62]. It appears that floristic barcode surveys at regional levels that use a local barcode library may provide an excellent tool for quick and reliable species identification. This includes many examples such as biodiversity monitoring, identification of plants that are prohibited from trading, authentication of medicinal plants collected from a region or auditing timber for illegal substitution with rare species of trees.

Commercial harvesting of timber is one of the major threats for its biodiversity in the TDEF of India. The threat is more prominent in case of the rare tree species that are listed in CITES Appendix II [78]. Though trading them within or outside the country is banned, their commercial value does attract illegal trading, which is well documented in the TDEF. Currently, it is very difficult to gather evidence and prosecute illegal trade of rare tree species. For example, the wood of *Santalum album* and *Osyris lanceolata* are anatomically similar which could not be distinguished easily [79]. A DNA barcode could serve as legal evidence of species identity from the traded parts of the plants, which is critical for supporting legal action against fraudulent or illegal trading. We utilized the TDEF reference barcode library that was developed from the current study to identity wood samples from commercial timber operations in the TDEF. We were able to identify 21 of timber samples at the species level, and the remaining 4 were identified at the genus level (Table 3). Although we only provide here a small case study, this does provide proof in principle that the TDEF reference barcode library could be used to more thoroughly audit timber operations throughout the TDEF. The 16 threatened species in the TDEF that are on the IUCN red list could be monitored using our TDEF reference barcode library, which provides legal evidence of enforcing conservation measures in the TDEF. This barcode library could be used to address the unsustainable and indiscriminate collection of plants from the wild for their medicinal value; 77 out of 143 tree species are traded as herbal remedies of which 28 are in high demand because they are highly effective in the commonly used traditional remedies [80]. In this case, the TDEF reference barcode library would also be useful for the authentication of commercial medicinal plant products, which are often adulterated (product substitution or contamination) with other species [81].

**Table 3:** Species identification of the logged timbers using TDEF reference barcode library.

S. No.	Sample ID	Vernacular name on the label	Scientific name	Species ID by DNA barcoding
1	TBW001	Poovarasu	*Thespesia populnea*	*Thespesia populnea*
2	TBW002	Sensandhanam	*Pterocarpus santalinus*	*Pterocarpus santalinus*
3	TBW003	Konravagai	*Peltophorum pterocarpum*	*Peltophorum pterocarpum*
4	TBW004	Anikundumani	*Adenanthera pavonina*	*Adenanthera pavonina*
5	TBW005	Cimaivagai	*Albizia saman*	*Albizia sp.*
6	TBW006	Nuna	*Morinda pubescens*	*Morinda pubescens*
7	TBW007	Mahogany	*Swietenia macrophylla*	*Swietenia macrophylla*
8	TBW008	Puliyamaram	*Tamarindus indica*	*Tamarindus indica*
9	TBW009	Saundal	*Leucaena latisiliqua*	*Leucaena latisiliqua*
10	TBW010	Pongum	*Pongamia pinnata*	*Pongamia pinnata*
11	TBW011	Thailam	*Eucalyptus tereticornis*	*Eucalyptus tereticornis*
12	TBW012	Theku	*Tectona grandis*	*Tectona grandis*
13	TBW013	Manja Kondrai	*Senna siamea*	*Senna siamea*
14	TBW014	Vembu	*Azadirachta indica*	*Azadirachta indica*
15	TBW015	Kattu Vaagai	*Albizia lebbeck*	*Albizia sp.*
16	TBW016	Mara Kumizh	*Gmelina arborea*	*Gmelina arborea*
17	TBW017	Pala	*Artocarpus heterophyllus*	*Artocarpus heterophyllus*
18	TBW018	Netilingam	*Polyalthia longifolia*	*Polyalthia longifolia*
19	TBW019	Purasu	*Butea monosperma*	*Butea monosperma*
20	TBW020	Iluppai	*Madhuca longifolia*	*Madhuca longifolia*
21	TBW021	Vilam Palam	*Limonia acidissima*	*Feronia limonia*
22	TBW022	Naval	*Syzygium cumini*	*Syzygium cumini*
23	TBW023	Velvel	*Acacia leucophloea*	*Acacia sp.*
24	TBW024	Sisu	*Dalbergia sissoo*	*Dalbergia sissoo*
25	TBW025	Karuvel	*Acacia nilotica*	*Acacia sp.*

## Supporting Information

Figure S1 NJ tree of TDEF reference barcode library for *rbcL* marker from 143 tree species.
(TIF)

Figure S2 NJ tree of TDEF reference barcode library for *matK* marker from 117 tree species.
(TIF)

Table S1 Collection sites from Tropical Dry Evergreen Forest (TDEF).
(XLSX)

Table S2 Details of the 429 samples collected for the present study, their process IDs in BOLD, PCR success and length of *rbcL* and *matK* sequences obtained.
(XLSX)

Table S3 Inter-specific divergence between congeneric species pairs for *rbcL* marker.
(XLSX)

Table S4 Inter-specific divergence between congeneric species pairs for *matK* marker.
(XLSX)

Table S5 Number and positions of diagnostics single pure CAs (sPu) for the TDEF tree species from *rbcL* marker.
(XLSX)

Table S6 Number and positions of diagnostics single pure CAs (sPu) for the TDEF tree species from *matK* marker.
(XLSX)
