# Supplementary figures and images for: DNA Barcode Authentication of Wood Samples of Threatened and Commercial Timber Trees within the Tropical Dry Evergreen Forest of India
Source: PLoS One. 2014 Sep 26;9(9):e107669. doi: 10.1371/journal.pone.0107669 (PMC4178033; doi:10.1371/journal.pone.0107669)

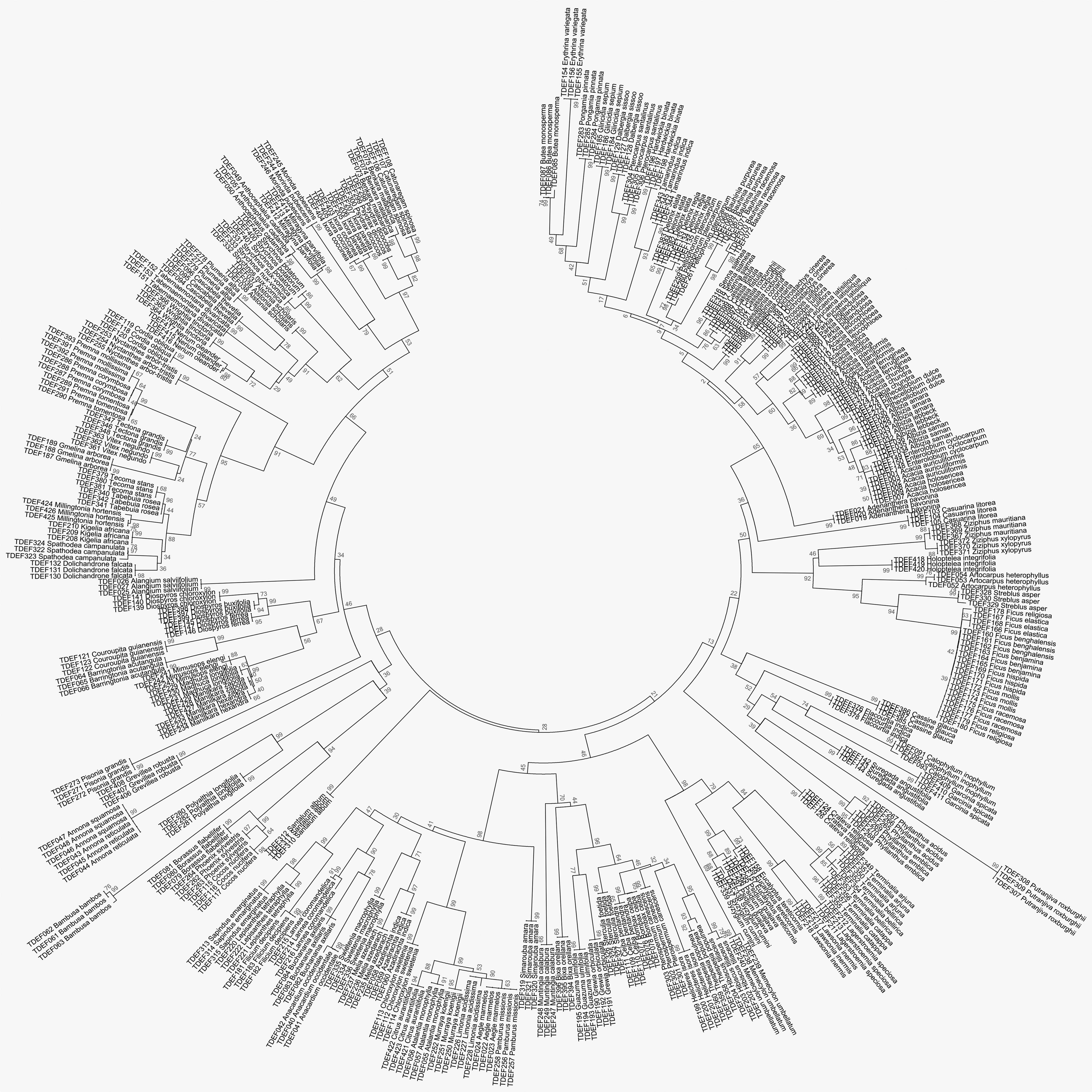

Supplement: Figure S1 — NJ tree of TDEF reference barcode library for rbcL marker from 143 tree species. (TIF) [file pone.0107669.s001.tif]

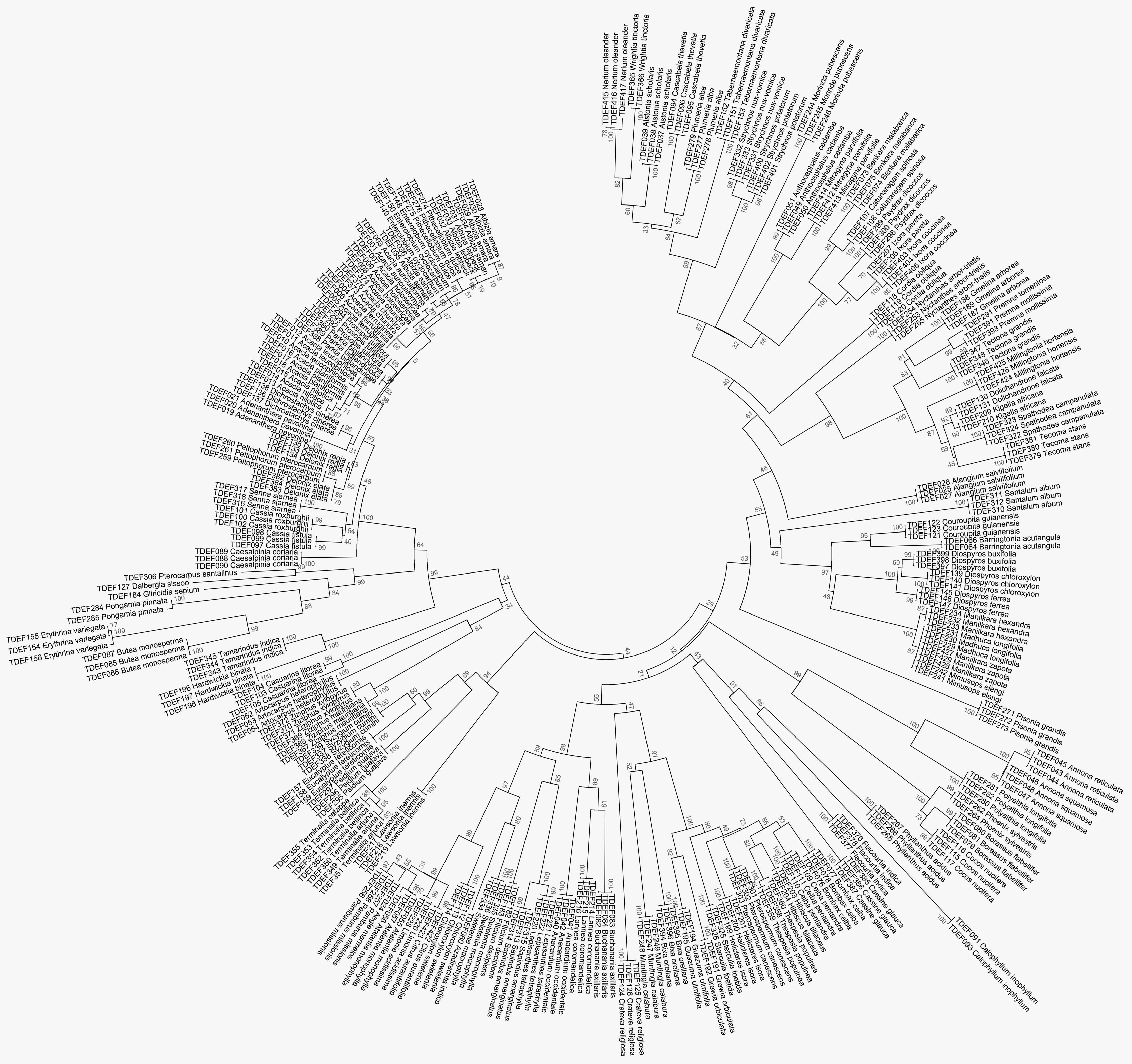

Supplement: Figure S2 — NJ tree of TDEF reference barcode library for matK marker from 117 tree species. (TIF) [file pone.0107669.s002.tif]
